# Bilateral carotid and vertebral rete mirabile with aneurysm misdiagnosed as Moyamoya disease: A case report

**DOI:** 10.1097/MD.0000000000039979

**Published:** 2024-10-11

**Authors:** Xiuen Chen, Chao Xiao, Chao Qin

**Affiliations:** a Department of Neurology, Liuzhou People’s Hospital Affiliated to Guangxi Medical University, Liuzhou, Guangxi, China; b Department of Neurology, The First Affiliated Hospital of Guangxi Medical University, Liuzhou, Guangxi, China.

**Keywords:** intracranial aneurysm, Moyamoya disease, rete mirabile

## Abstract

**Rationale:**

Rete mirabile (RM) is rare in humans, normally existing in lower mammalian arteries. To study its clinical and imaging features, we report an extremely rare case that occurred in humans and review the literature.

**Patient concerns:**

A 43-year-old female patient was admitted to our hospital because of recurrent dizziness and headache for 4 years.

**Diagnoses:**

The computed tomography angiography (CTA) of the head and neck indicated that the normal structure of the bilateral cervical and vertebral arteries disappeared, and the microvascular network formed, misdiagnosed as Moyamoya disease.

**Interventions:**

She underwent cerebral digital subtraction angiography examination and was finally diagnosed as carotid and vertebral RM with aneurysm.

**Outcomes:**

Following the administration of symptomatic treatment, all of her symptoms dissipated, and she was successfully discharged from the hospital.

**Lessons:**

RM involving in both anterior and posterior circulation is an extremely rare clinical abnormality of cerebrovascular morphology. The radiologists and clinicians should deepen their awareness of the specific CTA feature of RM. When individual CTA examination is insufficient, the utility of digital subtraction angiography is crucial for making a clear diagnosis.

## 1. Introduction

Rete mirabile (RM) represents an anomalous network of blood vessels that supplants a main artery affected by dysplasia or hypoplasia.^[1]^ This phenomenon is exceedingly rare in the human population but is a prevalent feature in the arterial systems of certain lower mammals. In human cases, the carotid RM is typically found within the cavernous sinus, while the vertebral RM is more commonly encountered at the foramen magnum. In both instances, the standard anatomical structure is supplanted by a complex system of collateral vessels.^[2,3]^ Predominantly, RM is associated with the carotid RM rather than the vertebral RM in isolation, and the co-occurrence of both is extraordinarily rare. A review of the existing literature reveals a mere handful of cases approximately 10 documenting the simultaneous presence of CVRM. These rare occurrences underscore the need for heightened clinical awareness and meticulous diagnostic approaches to ensure accurate identification and appropriate management of such vascular anomalies.^[4]^

Moyamoya disease is an idiopathic connective tissue disorder characterized by the insidious narrowing of the cerebral basal arteries, leading to a progressive occlusion. This condition is further distinguished by the compensatory development of a network of abnormal collateral vessels around the base of the skull. The term “moyamoya,” which translates to “puff of smoke” in Japanese, aptly describes the hazy, cloud-like appearance of these collateral vessels on cerebral angiography, a visual that has become emblematic of the disease’s unique vascular signature.^[5]^

This article presents a case study of a patient with CVRM accompanied by an intracranial aneurysm, which was initially misdiagnosed as Moyamoya disease. The patient’s imaging results bore a striking resemblance to those characteristic of Moyamoya disease, including occlusion of the internal carotid artery and the presence of abnormal vascular networks. The rarity of RM led to its initial misidentification as Moyamoya disease, a misdiagnosis that stemmed from a lack of clinical familiarity with RM’s manifestations. However, upon thorough review of the existing literature and a reevaluation of the patient’s vascular anomalies, the diagnosis was corrected to RM. This case highlights the importance of considering rare conditions in differential diagnoses, especially when imaging findings are ambiguous or overlap with more common conditions. It underscores the necessity for radiologists and clinicians to be aware of the diverse presentations of cerebrovascular diseases and the value of continuous medical education in refining diagnostic acumen.

## 2. Case presentation

A 43-year-old female was admitted to the hospital with a chief complaint of “recurrent dizziness and headaches over the past 4 years.” She reported a medical history of appendicitis 10 years prior, for which she had undergone surgical intervention. Additionally, she mentioned a 1-year history of sleep disorders. She denied any history of hypertension, diabetes, coronary heart disease, autoimmune disorders, or genetic conditions. Upon admission, her physical examination was found to be normal. Laboratory tests revealed elevated total cholesterol and low-density lipoprotein levels, along with mild anemia. However, her blood glucose, electrolytes, coagulation profiles, erythrocyte sedimentation rate, high-sensitivity C-reactive protein, anti-O, rheumatoid factor, and other relevant tests were all within the normal range.

**Figure 1.**
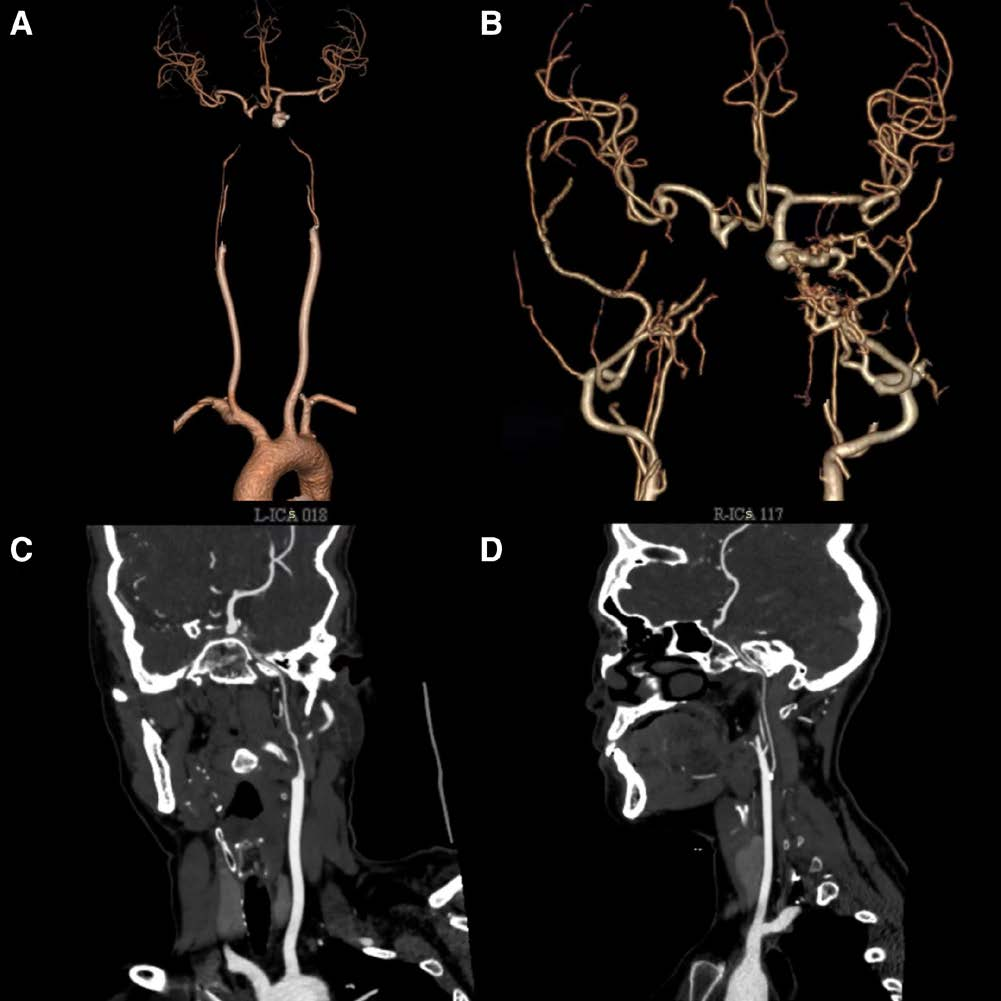
CT angiography showing bilateral internal carotid arteries were small and dysplasia, main trunks disappeared at the cavernous sinus. CT angiography = computed tomography angiography.

**Figure 2.**
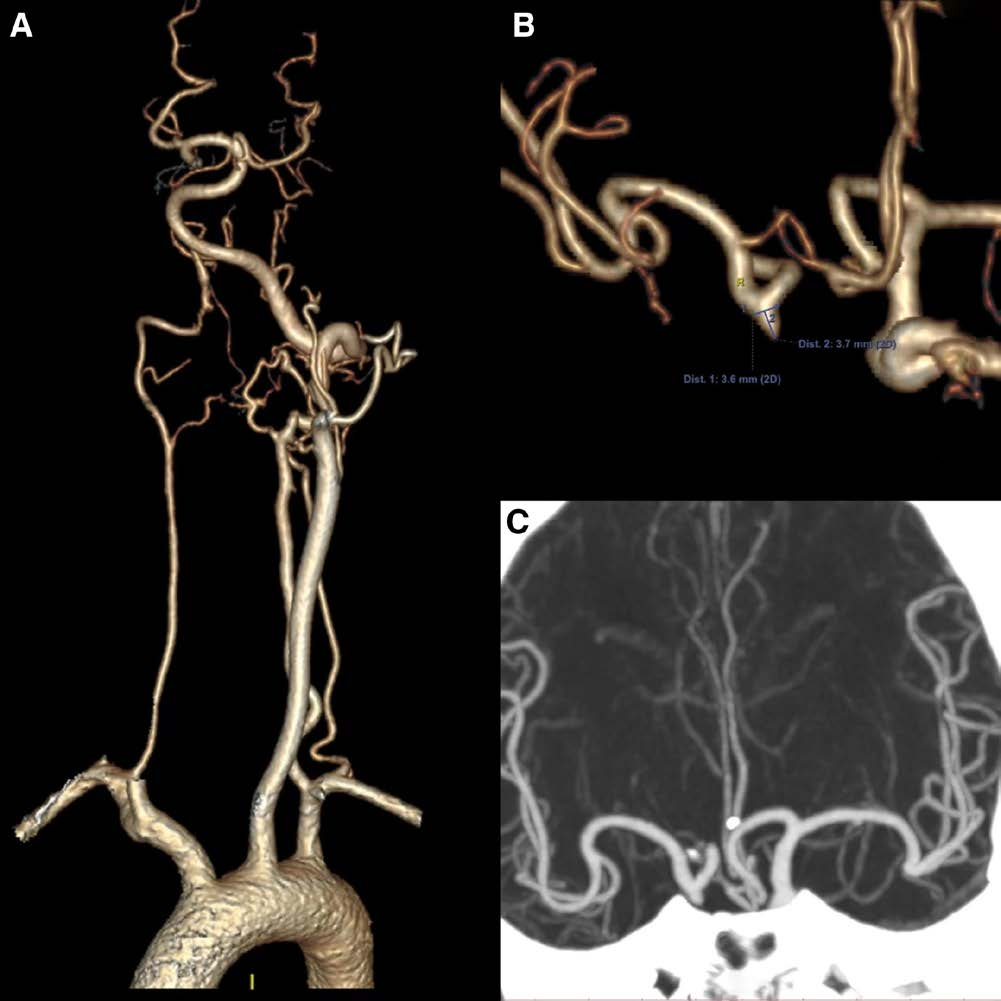
CT angiography showing that the right vertebral artery was slightly small, and the left vertebral artery was dominant. Main trunks disappeared at the foramen magnum and formed microvascular networks supplying blood to the skull (A). The left deep carotid artery formed a microvascular network at the foramen magnum and merged into the left vertebral artery (A).The vertebra-basal artery was dilated and tortuous (B). An aneurysm formed in the posterior traffic segment of the right internal carotid artery (C). CT angiography = computed tomography angiography.

A color Doppler ultrasonography examination of the patient’s cerebrovascular and cervical blood vessels revealed no significant abnormalities in blood flow velocity, spectral waveform, pulsatility index, or audio frequency within both the intracranial and cervical vasculature. Computed tomography angiography demonstrated that the bilateral internal carotid arteries were diminished in size and exhibited dysplasia, with the main trunks disappearing at the level of the cavernous sinus (Fig. 1). Additionally, an aneurysm was identified in the posterior communicating segment of the right internal carotid artery (Fig. 2).

The left vertebral artery was noted to be dominant, while the right vertebral artery appeared small. Multiple tortuous vascular shadows were observed surrounding the V4 segments of the vertebral arteries on both sides, with particular emphasis on the left vertebral artery–basilar artery, which was found to be tortuous. An aortic arch angiogram indicated that the branches were normal in appearance (Fig. 2).

**Figure 3.**
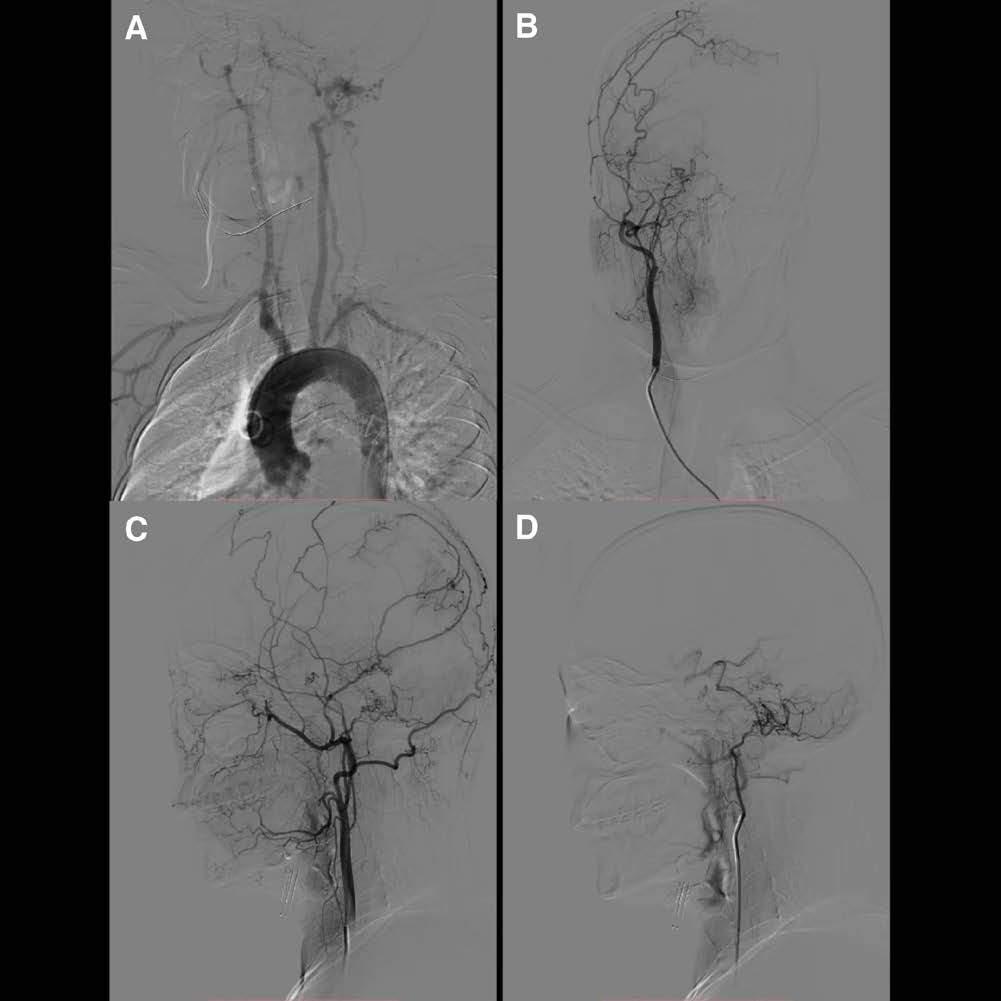
The aortic arch angiogram showing that branches were normal (A). The right common carotid artery orthotropic and lateral angiography showing that internal carotid artery was dysplasia, forming a microvascular network after main trunk disappearing (B and C). The internal maxillary artery also formed a microvascular network (C). The right posterior auricular artery lateral angiography showing that the formation of RM (D). RM = rete mirabile.

**Figure 4.**
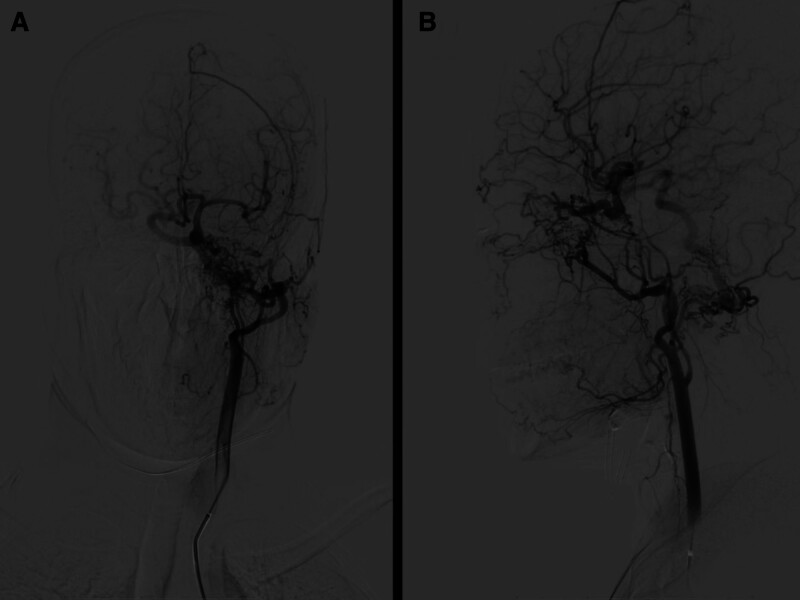
The left common carotid artery orthotropic and lateral angiography showing that internal carotid artery was dysplasia and formed a microvascular network after main trunk disappearing. The internal maxillary artery also formed a microvascular network and passed through the eye supplying blood to the brain with compensation. The ophthalmic artery was thickened and enlarged. Microvascular networks formed at the end of the occipital artery.

The bilateral internal carotid arteries exhibited a uniformly small and dysplastic appearance throughout their course. Upon reaching the cavernous sinus, the main trunks vanished, giving rise to a network of blood vessels (Figs. 3 and 4). External angiography revealed that the internal maxillary arteries had branched out to provide compensatory blood supply to this vascular network (Fig. 3). The left ophthalmic artery appeared thickened and enlarged (Fig. 4), while the left occipital artery and the right posterior auricular artery were part of the network. The right superficial temporal artery’s branches supplied blood to the intracranial region in a compensatory manner.

**Figure 5.**
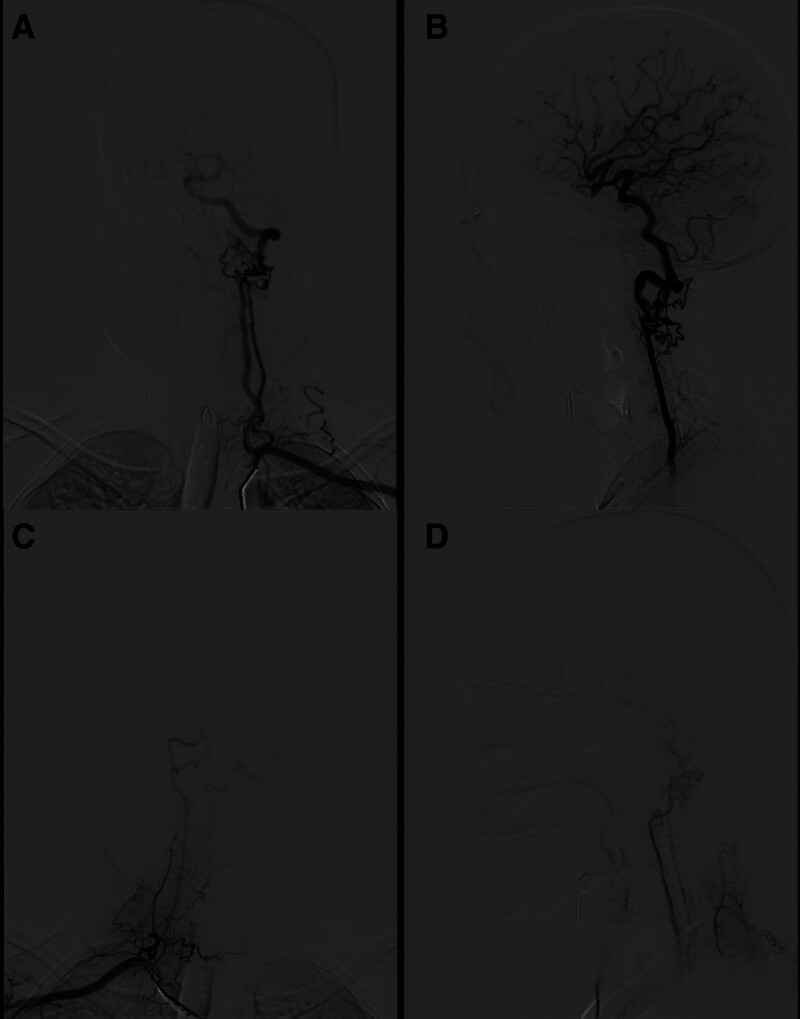
The left subclavian artery orthotropic and lateral angiography showing that the left vertebral artery RM formed, the left dep carotid artery RM formed (A and B). The vertebral–basal artery was tortuous and expanded (B). The right subclavian artery orthotropic and lateral angiography showing that the right vertebral artery RM formed (C and D). RM = rete mirabile.

Bilateral vertebral angiography demonstrated that the left vertebral artery was dominant, with the right vertebral artery being slightly smaller. The main trunks of the vertebral arteries on both sides disappeared in the region of the foramen magnum, where they continued as RM. A vascular network at the terminus of the left deep cervical artery was observed, which supplied blood to the left vertebral artery. The intracranial segments of the left vertebral artery base and the basilar artery were noted to be dilated and tortuous (Fig. 5).

**Figure 6.**
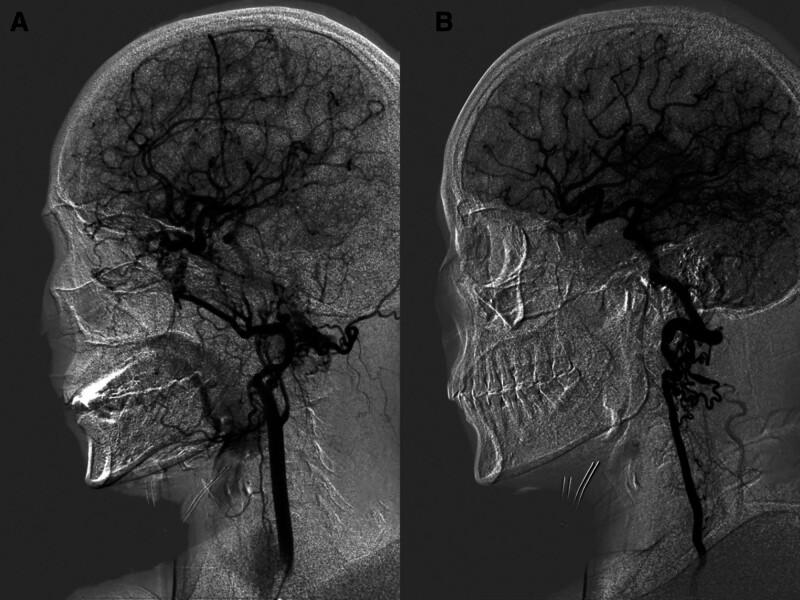
The left vertebral artery 3D angiography showing that the right posterior communicating artery enlarged and supplied blood to the right middle cerebral artery areas, and an aneurysm formed at the end of the right internal carotid artery.

Three-dimensional angiography of the left vertebral artery showed that the right posterior communicating artery had enlarged, supplying blood to the regions of the right middle cerebral artery, and an aneurysm was present at the terminus of the right internal carotid artery (Fig. 6).

Given the disease’s characteristics and the aforementioned imaging findings, the patient was diagnosed with a bilateral cervical and vertebral artery microvascular network accompanied by a right internal carotid aneurysm. The intracranial aneurysm was asymptomatic; therefore, a strategy of clinical observation was adopted for her condition. Following symptomatic treatment, the patient experienced an improvement in her dizziness and was subsequently discharged. It is planned to conduct regular follow-ups on the status of the intracranial aneurysms using computed tomography angiography or digital subtraction angiography of the head and neck.

## 3. Discussion

RM, also known as the intracranial microvascular network, serves as a secondary collateral circulation mechanism. It ensures the maintenance of intracranial blood flow when the primary artery is compromised and unable to provide adequate blood supply.^[6]^ RM is a normal anatomical feature in lower mammals, including but not limited to pigs, cats, and sheep, and it plays a crucial physiological role.^[7]^ It is responsible for maintaining blood flow and temperature regulation within the skull of these animals. In the early stages of mammalian embryonic development, the internal carotid or vertebral artery maintains continuity. However, as the embryo matures, these arteries undergo secondary atrophy and degeneration, culminating in the formation of a vascular network at the affected site.^[8]^ Certainly, in the normal course of human embryonic development, RM is not present. The etiology behind the occurrence of RM in humans remains elusive. According to the literature, there have been approximately 30 reported cases of RM worldwide. The age at diagnosis ranges from the youngest case at 13 years old to the oldest at 70 years old. The majority of these cases have been among Asian populations, predominantly involving the carotid RM, with only a few instances of combined vertebral RM, referred to as CVRM.^[4]^ The simultaneous occurrence of CVRM on both cerebrovascular vessels is exceedingly rare, and to date, there have been no reports documenting RM involving the occipital artery and the posterior auricular artery.

A comprehensive review of the literature on RM reveals that the clinical manifestations in patients are not distinctive. The primary symptoms encompass a range of presentations, including dizziness, balance disorders, subarachnoid hemorrhage, limb weakness, arteriovenous fistulas, and conditions resembling Pseudoxanthoma elastica, among others.^[4]^ Young patients appear to be at a higher risk for stroke. Lu et al^[9]^ have previously reported a case involving bilateral CVRM that presented with bilateral temporal pain. Our patient exhibited similar symptoms, with the pain manifestation not limited to dizziness but also including binocular pain and blurred vision. We considered that the patient’s symptoms are associated with the hemodynamic alterations induced by RM. The persistent patency of the secondary collateral circulation could potentially allow microemboli to access the retina or the intracranial space, thereby precipitating the observed symptoms. Additionally, the ocular swelling and pain might be attributed to an increase in collateral circulation, which in turn raises local blood flow and results in vasodilation.^[10]^ There have been relatively few documented cases of RM co-occurring with intracranial aneurysms. In a notable instance from 2015, Eric et al^[4]^ described a case involving RM accompanied by an ocular aneurysm, which was successfully managed through surgical intervention with craniotomy and clipping. In 2013, Morio^[11]^ and colleagues reported a case of bilateral CVRM that was associated with an anterior spinal cord aneurysm. Similarly, in 1996, Herwadkar^[11]^ and colleagues described a case of RM combined with a basilar artery apex aneurysm. In both instances, the aneurysms were effectively managed through endovascular coil embolization.

The genesis of an aneurysm is predominantly linked to hemodynamic alterations, particularly shifts in the blood vessel’s wall shear stress and wall shear stress gradient.^[12]^ In the present case, the left ophthalmic artery exhibited thickening and local dilation, and an aneurysm was observed at the terminus of the right internal carotid artery. It is hypothesized that the occurrence of RM may have led to an increase in blood flow, potentially contributing to the aneurysm formation.

The diagnosis of RM is not difficult, mainly based on the following characteristics of imaging^[13–15]^: (1) dysplasia of the internal carotid artery is present; (2) a vascular network exists between the maxillary artery and the cavernous sinus of the internal carotid artery; (3) the ophthalmic artery is observed to be enlarged; (4) when the internal carotid artery is obstructed, blood supply is maintained by the vascular network and the ophthalmic artery, with other segments showing good development; (5) bilateral lesions are noted; (6) there are no abnormal blood vessels characteristic of Moyamoya disease. Additionally, it is crucial to differentiate this condition from other cerebrovascular disorders, with a particular emphasis on distinguishing it from Moyamoya disease. Moyamoya disease is characterized by the progressive occlusion of bilateral internal carotid arteries and the development of collateral vascularization, which bears a resemblance to the manifestations of RM. Both conditions can present with stroke-like symptoms. Cerebrovascular angiography stands as the gold standard for definitive diagnosis, enabling the differentiation between these similar clinical pictures.^[16]^ Our patient was initially misdiagnosed with Moyamoya disease. Recognizing the imaging features of RM should not be challenging once familiar with its characteristics. The primary cause of this misdiagnosis is the clinical rarity of RM and the limited awareness among physicians. Upon establishing a clear diagnosis of RM, it is equally important to screen for the presence of any associated conditions in these patients, such as aneurysms, arteriovenous fistulas, arteriovenous malformations, Pseudoxanthoma elasticum, PHACE syndrome, and other related disorders. In this case, the patient exhibited the aforementioned imaging characteristics, leading to a definitive diagnosis. Additionally, the presence of an intracranial aneurysm was identified. Digital subtraction angiography revealed that RM was also affecting the occipital artery and the posterior auricular artery, marking this as the first reported instance of such involvement. Nearly all articles are presented as case reports. Future research will focus on exploring molecular markers to better understand this rare disease and its predictive factors.

In summary, RM is an uncommon clinical abnormality in cerebrovascular morphology, with CVRM being particularly rare. The clinical signs and symptoms of RM are nonspecific, which makes it prone to misdiagnosis. To enhance understanding of the pathogenesis and clinical features.

## Author contributions

**Project administration:** Chao Qin.

**Validation:** Chao Xiao.

**Writing – original draft:** Xiuen Chen.

**Writing – review & editing:** Xiuen Chen.
